# Suppressed sympathetic outflow to skeletal muscle, muscle thermogenesis, and activity energy expenditure with calorie restriction

**DOI:** 10.14814/phy2.13171

**Published:** 2017-02-27

**Authors:** Tariq I Almundarij, Chaitanya K. Gavini, Colleen M Novak

**Affiliations:** ^1^ College of Agriculture and Veterinary MedicineAl Qassim UniversityBuraydahAl‐Qassim ProvinceSaudi Arabia; ^2^ Department of Biological SciencesKent State UniversityKentOhio; ^3^ Department of Cell and Molecular PhysiologyStritch School of MedicineLoyola University ChicagoMaywoodIllinois; ^4^ School of Biomedical SciencesKent State UniversityKentOhio

**Keywords:** melanocortin 4 receptor (MC4R), nonexercise activity thermogenesis (NEAT), norepinephrine turnover (NETO), sympathetic nervous system (SNS), ventromedial hypothalamus (VMH)

## Abstract

During weight loss, adaptive thermogenesis occurs where energy expenditure (EE) is suppressed beyond that predicted for the smaller body size. Here, we investigated the contributions of resting and nonresting EE to the reduced total EE seen after 3 weeks of 50% calorie restriction (CR) in rats, focusing on activity‐associated EE, muscle thermogenesis, and sympathetic outflow. Prolonged food restriction resulted in a 42% reduction in daily EE, through a 40% decrease in resting EE, and a 48% decline in nonresting EE. These decreases in EE were significant even when the reductions in body weight and lean mass were taken into account. Along with a decreased caloric need for low‐to‐moderate‐intensity treadmill activity with 50% CR, baseline and activity‐related muscle thermogenesis were also suppressed, though the ability to increase muscle thermogenesis above baseline levels was not compromised. When sympathetic drive was measured by assessing norepinephrine turnover (NETO), 50% CR was found to decrease NETO in three of the four muscle groups examined, whereas elevated NETO was found in white adipose tissue of food‐restricted rats. Central activation of melanocortin 4 receptors in the ventromedial hypothalamus stimulated this pathway, enhancing activity EE; this was not compromised by 50% CR. These data suggest that suppressed activity EE contributes to adaptive thermogenesis during energy restriction. This may stem from decreased sympathetic drive to skeletal muscle, increasing locomotor efficiency and reducing skeletal muscle thermogenesis. The capacity to increase activity EE in response to central stimuli is retained, however, presenting a potential target for preventing weight regain.

## Introduction

As the obesity epidemic progresses, there is a greater push to encourage weight loss, commonly through low calorie diets. With the new, lower body weight comes decreased energy need. Perhaps the most insidious result of successful dieting is the adaptive thermogenesis that accompanies reduced body weight (Rosenbaum and Leibel 2010). In this phenomenon, energy expenditure (EE) decreases beyond what is predicted for the lower body mass (Rosenbaum and Leibel 2010; Dulloo et al. 2012; Muller and Bosy‐Westphal 2013). This has the effect of countering further weight loss or weight maintenance efforts, undermining the long‐term success of the diet (Tremblay et al. 2013; Fothergill et al. 2016).

Adaptive thermogenesis after food restriction has been documented under different conditions in laboratory animals (Ravussin et al. 2011) and in humans, and at least part of this adaptation occurs in nonresting EE, particularly in activity thermogenesis (Rosenbaum et al. 2003; Goldsmith et al. 2010; Muller and Bosy‐Westphal 2013; Rosenbaum and Leibel 2016). While maintaining a 10% weight reduction after calorie restriction (CR), patients experience an increase in skeletal muscle work efficiency (Rosenbaum et al. 2003)—using fewer calories to perform the same work, even after the lower body mass is taken into account (Rosenbaum et al. 2003). Here, we use a rat model to quantify the contribution of adaptive thermogenesis occurring in the energy expenditure of low‐to‐moderate‐intensity physical activity, termed nonexercise activity thermogenesis (NEAT) in humans. Individual differences in NEAT are predictive of fat gain in response to overfeeding (Levine et al. 1999), and NEAT is higher in lean compared to obese individuals (Levine et al. 2005). Here, we measure rat spontaneous locomotor activity and locomotor efficiency along with activity thermogenesis *per se* – the heat dissipated by skeletal muscle during activity, an indicator of muscle energy inefficiency.

Human adaptive thermogenic responses in the nonresting and resting compartments of EE follow different trajectories, where nonresting EE is more susceptible to further suppression with additional weight loss, even in the absence of negative energy balance (Rosenbaum and Leibel 2016). This implicates different or additional mechanisms impacting nonresting EE. Adaptations in skeletal muscle energetic mechanisms are likely to contribute more to nonresting than resting EE; though skeletal muscle oxygen uptake is an important determinant of resting metabolic rate (Zurlo et al. 1990), changes in muscle energetics are directly associated with altered fuel economy during physical activity (Rosenbaum et al. 2005; Goldsmith et al. 2010; Novak et al. 2010; Baldwin et al. 2011; Gavini et al. 2014). While adjustments in sympathetic nervous system (SNS) activity, including SNS effects on muscle, have been proposed as mechanisms underlying human adaptive thermogenesis (Aronne et al. 1995; Rosenbaum et al. 2000), to the best of our knowledge, suppressed SNS drive explicitly to skeletal muscle has not been reported. Here, we investigate altered muscle work efficiency during CR, hypothesizing that SNS outflow to muscle is suppressed during energy restriction. Previously, measuring norepinephrine (NE) turnover (NETO) to assess SNS drive, dietary manipulation was found to impact brown adipose tissue and heart but not skeletal muscle; (Dulloo et al. 1988) in this case, the food restriction consisted of a 2‐day fast. The effects of energy restriction on physical activity and activity‐related EE, however, depend on the duration of the restriction (Novak et al. 2012). Short‐term restriction (1–3 days) increases physical activity,(Smyers et al. 2015) presumably a foraging response in laboratory rodent models. Prolonged restriction (weeks) clearly suppresses physical activity and increases fuel economy of activity (muscle work efficiency), promoting energy conservation, with individual differences in the speed of these adaptations (Smyers et al. 2015). Here, we investigate CR‐induced changes in NETO in skeletal muscle during negative energy balance after several weeks of food restriction in rats.

When considering potential central modulators of autonomic control of metabolism, the brain melanocortin system is a likely contributor, partly because it is an important mediator of leptin and leptin's effects on energy balance (Kim et al. 2014). While leptin is one important factor modulating adaptive thermogenesis, other aspects of adaptation to energy restriction are not accounted for by leptin (Rosenbaum et al. 2002, 2005; Rosenbaum and Leibel 2010; Baldwin et al. 2011; Camps et al. 2015; Lage et al. 2016). Genetic alterations in melanocortin peptides, their receptors, and their upstream and downstream modulators are commonly identified as contributors to human obesity, both monogenic and polygenic (Zegers et al. 2012). The melanocortin 4 receptor (MC4R) is particularly relevant to human obesity,(Loos 2011) and it is also an important regulator of autonomic control of metabolism.(Sohn et al. 2013; Berglund et al. 2014) Using a mixed receptor agonist, we have found that activation of ventromedial hypothalamic (VMH) melanocortin receptors increases energy use during activity, along with muscle thermogenesis and SNS drive to muscle (Gavini et al. 2016). Altogether, this evidence implicates altered melanocortin receptor function in the metabolic changes seen during altered fuel availability, especially with respect to skeletal muscle energy use. Here, we investigate the role of MC4R in the modulation of muscle work efficiency, and test the hypothesis that energy restriction alters economy of activity through decreasing the response to central activation of MC4R.

## Materials and Methods

### General methods

Adult, male Sprague‐Dawley rats (total *N* = 48) were selected to measure adaptive thermogenesis in a baseline population, though the argument has been made that even standard models are potentially metabolically morbid (Martin et al. 2010), reflecting the widespread incidence of overweight and obesity in the human population (Flegal et al. 2016). Rats were housed at 22 ± 1°C with minimal variation in ambient temperature on a 12:12 light:dark cycle with lights‐on at 0700 Eastern Standard Time. Rats were given ad libitum access to rodent diet (Lab Diet 5001; Lab Diet, Richmond, Indiana) before dietary intervention, and water throughout the studies. For 50% CR, body weight and food intake were measured for 7–8 consecutive days, and 50% food intake was calculated for each rat; this amount of food was given to rats for 21 days, and continued for additional days of CR necessary to complete energetic measurements. Body composition was measured using an EchoMRI‐700 (EchoMRI LLC, Houston, TX). At least 1 day before a rat underwent its first treadmill test, it was acclimated to the treadmill (10 min walk). All procedures and handling were in accordance with and approved by Kent State University's Institutional Animal Care and Use Committee.

### Surgery

Stereotaxic surgery was performed to implant guide cannulae aimed at the VMH (*N* = 15) as described previously (Shukla et al. 2012). Briefly, rats were placed in the stereotaxic apparatus after anesthesia with inhaled isoflurane. The following coordinates were used for VMH: anterior‐posterior, −2.5 mm; medial‐lateral, +0.5 mm; dorsal‐ventral, −6 mm (from bregma); and an injection needle with 3 mm projection (final dorsal‐ventral, −9 mm (from dura)). Guide cannulae were affixed to the skull using a sterile wound clip and dental cement, and rats were then allowed to recover before the start of the study. At the completion of the study, injection sites were determined histologically, and only rats with sites within 250 *μ*m of the VMH were included in the analyses.

For measurement of muscle thermogenesis before and after CR (*N* = 8), muscle temperature transponders (IPTT‐300, BioMedic Data Systems, Inc.) were implanted in both hind limbs adjacent to the gastrocnemius (gastroc) muscle group under isoflurane anesthesia as described previously (Gavini et al. 2014, 2016). Transponders did not disrupt locomotion and were not impacted by body composition measurement.

### Energy expenditure

Energy expenditure was measured (*N* = 8) before and after 3 weeks of 50% CR. Baseline (ad libitum‐fed) EE was measured using an Oxymax FAST system (Columbus Instrument, Columbus, OH) as described previously (Gavini et al. 2014). Body composition was measured before baseline calorimetry, and again on day 19 of CR. Rats were acclimated to the chamber (at thermoneutral conditions for rats, 25–26°C) and calorimetry cage for at least 24 h before measurement of gas exchange. The calorimeter was calibrated using primary gas standards, and air was supplied to the rats at 2.2‐2.7 LPM, depending on the size of the rat; sample flow was 0.5 LPM. Gas exchange was measured every 30 sec, with reference measurements taking place after every 30 samples. Physical activity levels were measured in the X and Z axes every 10 sec throughout the testing period. Average values were calculated starting at 1200 on day 1 through 1200 on day 2. For post‐CR measurements, calorimetry was started on day 23 and 24 of CR.

Before CR and on days 21‐22 of CR, treadmill‐activity EE was measured similarly using an enclosed treadmill attached to the calorimeter (Gavini et al. 2014, 2016). Rats were acclimated to the treadmill for 2 h without food, while gas exchange was being measured with 2.5‐2.6 LPM air flow to the treadmill. The rats then walked on the treadmill at 7 m/min for 30 min while gas exchange was measured every 10 sec.

### Muscle temperature

In the eight rats measured for EE (see section 2.3), gastroc muscle heat dissipation was measured over the course of a low‐to‐moderate‐intensity treadmill‐walking protocol, as described previously (Gavini et al. 2014, 2016). Activity‐related muscle heat dissipation was measured both before and after (on days 21–22) 50% CR. Briefly, an IPTT DAS‐7007s reader (BioMedic Data Systems) was used to measure temperature in each hind leg as rats completed a graded treadmill test that induced physical activity ranging from low‐intensity to moderate‐intensity: after 2, 5, and 10 min of 7 meters/min at 0° incline; at 9 m/min at 0° incline (at 15 min); at 9 m/min, 10° incline (at 20 min); at 11 m/min, 10° incline (at 25 min); at 11 m/min, 20° incline (30 and 35 min).

### Norepinephrine turnover (NETO)

NETO is used to assess SNS drive to peripheral tissues by factoring out pre‐ and postsynaptic effects (e.g., differences in reuptake), as described previously (Gavini et al. 2014). In this study, 25 rats were divided into two groups, with one group remaining on ad libitum feeding (*n* = 12) and the other subjected to 50% CR (*n* = 13). Within these groups, one‐half were assigned to receive the competitive NE synthesis inhibitor *α*‐methyl‐p‐tyrosine (aMPT; *n* = 6 ad libitum‐fed rats, *n* = 7 CR rats), while the remaining rats did not receive aMPT. On the day of the study (days 21–22 of 50% CR), aMPT (125 mg aMPT/Kg of BW, 25 mg/mL) was injected into the assigned group at each 4 and 2 h before being killed by rapid decapitation between 1100 and 1600 EST. Skeletal muscle groups removed included medial and lateral gastroc, quadriceps, and soleus; also collected were epididymal white adipose tissue (EWAT), liver, and heart but sufficient brown adipose tissue could not be accurately identified and isolated after CR. All tissues were rapidly frozen in liquid nitrogen. To isolate and measure catecholamines, thawed tissue was homogenized with the internal standard, dihydroxybenzylamine (DHBA) with 1 mg/mL ascorbic acid (AA) in 0.2mol/L perchloric acid (PCA), and centrifuged at 7500 *g* at 4°C for 15 min. Alumina was used to extract the catecholamine solution which was eluted with PCA/AA and assayed using high performance liquid chromatography with electrochemical detection (HPLC‐EC; Coulochem III) using MDTM mobile phase and a reverse phase MD 150 × 3.2 column. The following formula was used to calculate NETO, with a greater aMPT‐associated decrease in tissue NE reflected in higher NETO values: (Vaughan et al. 2014) *k * =  (lg[NE]0 – lg[NE]4)/(0.434 × 4); K  =  *k*[NE]0; *k* is the constant rate of NE efflux (also known as fractional turnover rate), [NE]0 is the initial NE concentration from 0‐hr group (control), [NE]4 is the final NE concentration from 4‐hr group (aMPT), and K  =  NETO. Body composition was measured in all rats before and on the 21st day of CR or control feeding.

### MC4R agonist microinjection

We used the potent and selective MC4R agonist Cyclo(b‐Ala‐His‐D·Phe‐Arg‐Trp·Glu)NH2 (Phoenix Pharmaceuticals)(Bednarek et al. 2000, 2001); the thermogenic effects of this agonist are blocked by a specific MC4R antagonist in Siberian hamsters (Vaughan et al. 2011), and this agonist induces changes in physical activity distinct from those induced by a specific melanocortin 3 receptor agonist in rats (Shukla et al. 2015). The MC4R agonist was microinjected into the VMH at the dose of 20pmoles/200 nL (200 nL volume, similar to the effective dose of the mixed melanocortin agonist melanotan II (Gavini et al. 2016) as previously described (Shukla et al. 2012). Rats (*N* = 15) were given microinjections of the MC4R agonist or vehicle (artificial cerebrospinal fluid, aCSF) on different days, in random order, both before CR and after 50% CR. After injection, rats were acclimated in an enclosed treadmill for 2 h without food, while gas exchange was measured with air supplied to the treadmill at 2.59–3.3 LPM, with reference air sampled every 30 min. Rats then walked on the treadmill at 7 meters/min for 30 min, as described above. Gas exchange was measured with air supplied to the treadmill at 2.59–3.3 LPM. Body composition was measured before CR and on the 19th day of 50% CR. After the conclusion of the study, brain sections were stained and examined for accuracy (microinjection sites not within 250 *μ*m of the VMH were eliminated from the analyses). Although the VMH appears to be the primary site of central modulation of skeletal muscle metabolism, (Shiuchi et al. 2009; Toda et al. 2009; Miyaki et al. 2011) it is possible that neighboring hypothalamic regions were also affected by the MC4R agonist and that this could also contribute to the thermogenic effects.

### Statistical analysis

Paired t‐tests (1‐tailed; predicted decrease in RER, activity, and temperature with CR) were used to compare respiratory exchange ratio (RER), activity variables, and baseline muscle temperatures before and after CR. Because of the dominant influence of body weight on EE, analysis of covariance (ANCOVA) was used to compare EE (kcal/hr) variables before and after CR, with body weight and lean mass as covariates in separate analyses. Muscle temperature during treadmill activity was analyzed using a repeated‐measures analysis of variance (ANOVA), with feeding condition and time on treadmill as the independent variables. NETO data were analyzed using two‐tailed t‐tests. Treadmill calorimetry data were analyzed using two‐way repeated‐measures ANOVAs, with covariance used when body weight and lean mass needed to be considered in the analysis (i.e., for EE comparisons before vs. after CR). NETO data were analyzed using unpaired t‐tests (two‐tailed) to compare unstimulated NETO values between ad libitum and 50% CR feeding conditions. Outliers were identified in heart (2 CR/AMPT values) and in liver (1 ad libitum/AMPT value) using a stringent criterion (>6 SEMs above the mean of all [NE]/gram sample weight for that tissue). Intra‐VMH MC4R agonist‐induced changes in treadmill‐calorimetric variables were compared using a 2 × 2 repeated‐measures ANOVA with the within‐subjects independent variables of vehicle versus agonist treatment, and before versus after CR. To rule out the confounding effect of body weight on EE, ANCOVA was used to analyze the effect of the MC4R agonist on EE.

## Results

### Calorie restriction induces adaptive thermogenesis in both resting and activity EE, increasing muscle work efficiency and decreasing muscle activity thermogenesis

**Figure 1:**
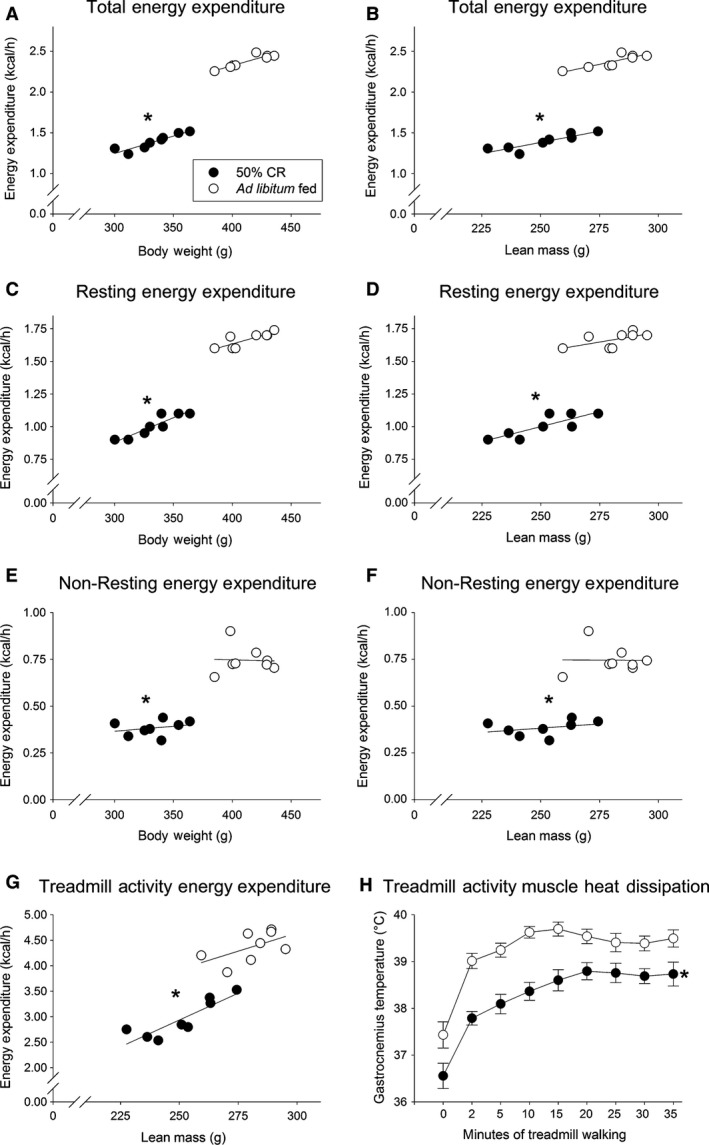
Three weeks of 50% calorie restriction (CR) significantly suppressed both resting and nonresting energy expenditure (EE), including physical activity‐related EE. Total EE (A–B), resting EE (C–D), and nonresting EE (E–F) were each significantly suppressed after CR when covariate analysis was used to factor out differences in body weight (A, C, E) or lean mass (B, D, F). When physical activity was controlled using a treadmill, there was a significant suppression in EE (G) as well as gastrocnemius muscle thermogenesis (H), though the increase above baseline in muscle thermogenesis at the higher intensities was not compromised by CR. *Significantly different from ad libitum‐fed rats, *P* < 0.05. (A–G: *N* = 8, each plotted under both conditions; H: *N* = 8 except for 35 min, where *N* = 6).

As shown in Table 1 3 weeks of 50% CR significantly reduced body weight and fat and lean mass. Accordingly, we saw a significant reduction in total EE (41.55% ± 1.06%) as well as in both resting EE (39.67% ± 1.36%) and nonresting EE (48.23%±2.44%); this persisted when either body weight or lean mass were taken into consideration using covariance (Fig. 1). Of the decrease in total EE, 35.14% (±1.47%) of this was from suppressed nonresting EE. Treadmill‐activity EE also significantly decreased after CR (by 31.21% ± 2.57%) with lean mass as the covariate (Fig. 1G); 24‐hr RER was significantly reduced (Table 1), as was treadmill‐activity RER (before CR, 0.86 ± 0.01; after CR, 0.79 ± 0.01). In summary, 50% CR induced marked suppression in weight‐corrected EE, including resting EE, nonresting EE, and EE during treadmill walking.

**Table 1:** Body composition, energy expenditure, and physical activity before and after 3 weeks of 50% calorie restriction (CR)

	BW (g)	Fat mass (g)	Lean mass (g)	*V*O_2_ (mL/kg/h)	*V*CO_2_ (mL/kg/h)	RER	Physical activity (counts/min)		
							Horizontal	Ambulatory	Vertical
Ad libitum	412 ± 7	33 ± 2	281 ± 4	1160 ± 8	1184 ± 7	0.93 ± 0.01	4.47 ± 0.18	1.97 ± 0.11	0.59 ± 0.06
50% CR	333 ± 7^a^	12 ± 2^a^	251 ± 5^a^	845 ± 9^a^	761 ± 10^a^	0.90 ± 0.01^a^	2.33 ± 0.09^a^	1.02 ± 0.04^a^	0.30 ± 0.02^a^

BW, body weight (g); *V*O_2_, volume of O_2_ consumed (ml/kg/hr); *V*CO_2_, volume of CO_2_ consumed (ml/kg/h).

a Significant change from ad libitum conditions, *P* < 0.05 (*N* = 8).

(Mean ± SEM).

Baseline average (left and right legs) gastroc temperatures were significantly lower after CR (36.6°C ± 0.3°C; paired t‐test, *P* < 0.01) compared to pre‐CR baseline temperatures of 37.4°C ± 0.3°C (similar to resting temperatures in the same rats at later, postexperimental body weights of 596.2g ± 12.9 g: 37.4°C ± 0.2°C). Within condition, muscle temperature was not positively correlated with body weight (i.e., there was no indication that muscle temperature was higher in heavier rats) either before CR (*r* = −0.339) or after CR (*r* = −0.028).

All rats finished the 35‐min graded treadmill test before CR, and six of the eight rats finished the test after CR, with the remaining two rats completing 30 min of the test. Gastroc temperature during treadmill activity averaged over both legs showed a significant interaction between dietary condition and time, where the baseline and activity‐associated muscle heat dissipation was suppressed by CR, with a lower magnitude of suppression as activity duration and intensity increased (Fig. 1H). Main effects of diet (CR) and time on treadmill were also seen. Compared to after CR, pre‐CR gastroc temperature was higher in each of the right and left legs at each time point except for the 35‐min time point in the left leg and the 2‐min time point in the right leg. Change in temperature from baseline (before treadmill was started) over the treadmill test showed a significant interaction where, in the 50% CR condition compared to free‐fed conditions, muscle temperature increased less toward the beginning of the treadmill‐walking protocol but increased more toward the end of the test. There was a main effect of time on treadmill, but not a main effect of CR in change from baseline temperature. These same main effects and interactions were seen when left or right gastroc temperatures were analyzed alone. In the right gastroc, change in temperature at 30 min was higher in the rats after CR, with a trend in the same direction between 20 and 35 min. In short, baseline and activity‐induced muscle temperature was lowered by CR, but the ability of moderate‐intensity physical activity to increase muscle thermogenesis was preserved, and the change in muscle temperature relative to baseline was significantly greater in the right gastroc on at least one time point. In other words, during treadmill walking, the rats could increase their muscle thermogenesis as much or more after CR as before CR, they just need to be more active after CR.

### Calorie restriction decreases SNS drive to skeletal muscle, while increasing NETO to white adipose tissue

**Figure 2:**
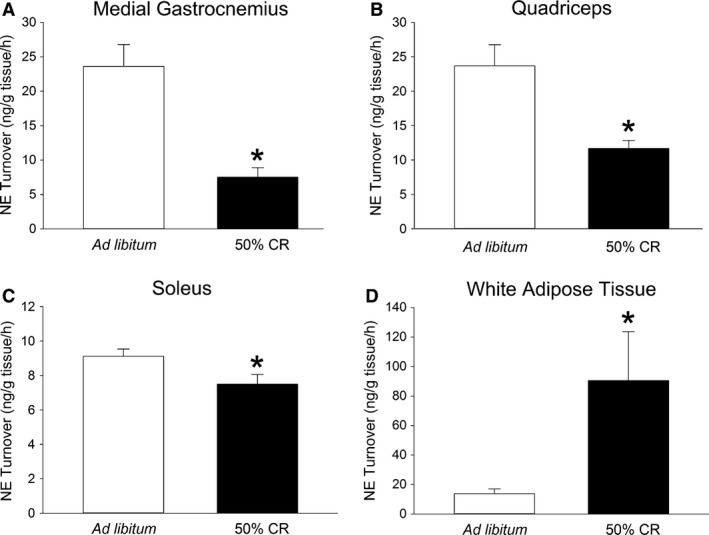
Daily calorie restriction of 50% (CR) for 3 weeks suppressed skeletal muscle norepinephrine turnover (NETO), an indicator of sympathetic nervous system drive. CR induced a significant decrease in NETO in (A) medial gastrocnemius, (B) quadriceps, and (C) soleus muscle groups, whereas 50% CR significantly increased NETO in epididymal white adipose tissue (D). *Significantly different from ad libitum‐fed rats, *P* < 0.05. (*N* = 6–7).

As shown in Figure 2 3 weeks of 50% CR significantly decreased NETO in the quad, soleus, and medial gastroc but not in the lateral gastroc (ad libitum, 31.45 ± 2.64 ng/g tissue/hr; CR, 31.71 ± 2.26 ng/g tissue/h). NETO was significantly elevated in EWAT after 50% CR. Compared to ad libitum conditions, NETO after 50% CR was significantly lower in heart (ad libitum, 21.67 ± 2.68 ng/g tissue/hr; CR, 6.71 ± 0.54 ng/g tissue/hr; *P* = 0.000334) with no change in liver (ad libitum, 64.31 ± 10.42 ng/g tissue/h; CR, 59.16 ± 6.46 ng/g tissue/h). Calorie‐restricted rats lost weight (from 419.9 g ± 7.6 g to 343.3 g ± 6.4 g), fat (from 39.0 g ± 2.2 g to 17.1 g ± 2.0 g), and lean mass (from 312.5 g ± 6.0 g to 271.2 g ± 4.9 g). This applied to the rats that received aMPT (*n* = 7) and to no‐aMPT controls (*n* = 5), with no significant differences between the groups. Over the same time period, ad libitum‐fed rats gained weight (from 412.5 g ± 4.8 g to 447.9 g ± 6.8 g), fat (from 36.6 g ± 2.0 g to 41.6 g ± 1.8 g), and lean mass (from 309.4 g ± 3.2 g to 333.3 g ± 6.3 g), with no significant differences between ad libitum‐fed aMPT rats (*n* = 7) and ad libitum‐fed no‐aMPT controls (*n* = 5). In short, rats subjected to 50% CR showed suppressed NETO in three out of four hind limb muscle groups and in heart but elevated NETO in EWAT.

**Figure 3:**
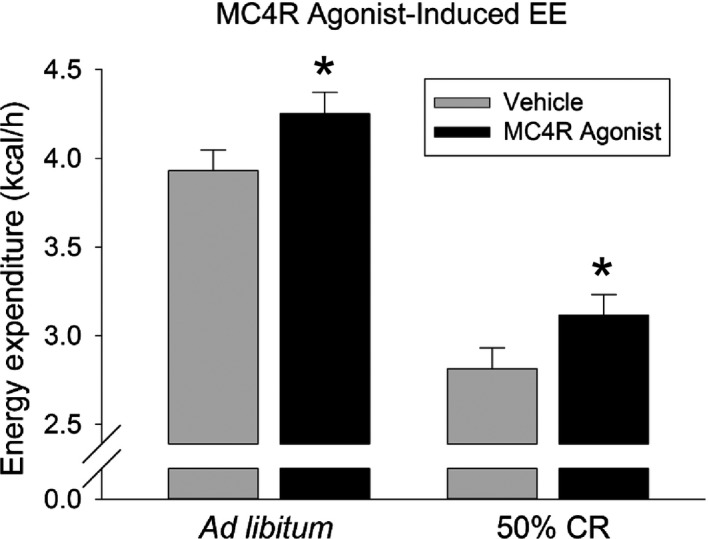
Daily calorie restriction of 50% (CR) did not compromise the ability of ventromedial hypothalamic (VMH) melanocortin 4 receptor (MC4R) activation to enhance activity energy expenditure (EE). Activation of MC4R by microinjections of the MC4R agonist (20pmoles/200 nL) into the VMH significantly increased the kcal used to walk on a treadmill at 7 meters/min for 30 min. After CR, the MC4R activation still significantly enhanced treadmill‐activity EE. There was no significant effect of 50% CR on the magnitude of this effect. *Significantly greater than vehicle, *P* < 0.05. (*N* = 8).

### The ability of VMH MC4R activation to increase activity EE is not compromised by calorie restriction

Eleven rats had correct cannula placement, and full datasets (treadmill EE for both ad libitum and CR conditions, and for both vehicle and MC4R agonist treatments) were collected for eight of these. As shown in Figure 3 and Table 2, intra‐VMH microinjections of the MC4R agonist induced significant increases in EE, *V*O_2_, and *V*CO_2_ (main effect of MC4R agonist) but not in RER. There was also a significant main effect of CR where 3 weeks of 50% CR significantly decreased EE, *V*O_2_, *V*CO_2_, and RER (Table 2). There were no significant interactions, that is, the ability of the MC4R agonist to alter treadmill‐activity EE did not change with CR. Using body weight as the covariate (significant effect of body weight on EE), there were significant main effects of the MC4R agonist and CR, where intra‐VMH MC4R microinjection significantly increased EE, and 3 weeks of 50% CR significantly decreased EE; again, there was no interaction. Three weeks of 50% CR significantly decreased body weight (from 403.1 g ± 5.9 g to 364.3 g ± 6.7 g), lean mass (from 309.5 g ± 4.8 g to 228.3 g ± 4.7 g), and fat mass (from 34.7 g ± 1.5 g to 12.5 g ± 1.1 g). When the vehicle‐stimulated treadmill EE was analyzed with body weight as the covariate, there was a significant effect of CR where treadmill EE was significantly lower after CR compared to before CR. In summary, central activation of MC4R augmented treadmill‐walking EE, and this effect remained even as EE dropped along with body weight with CR.

**Table 2:** Gas exchange variables before and after 3 weeks of 50% calorie restriction (CR) in rats treated with an MC4R agonist and vehicle in the ventromedial hypothalamus

	*V*O_2_ (mL/kg/h)		*V*CO_2_ (ml/kg/h)		RER (*V*CO_2_/*V*O_2_)	
	Ad libitum	50% CR^b^	Ad libitum	50% CR^b^	Ad libitum	50% CR^b^
MC4R agonist	2098 ± 60^a^	1823 ± 65^a^	1811 ± 50^a^	1469 ± 62^a^	0.864 ± 0.013	0.805 ± 0.009
Vehicle	1952 ± 120	1694 ± 59	1676 ± 41	1358 ± 55	0.859 ± 0.009	0.801 ± 0.008

(Mean ± SEM; *N* = 8 rats that completed all conditions).

*V*O_2_, volume of O_2_ consumed (ml/kg/hr); *V*CO_2_, volume of CO_2_ consumed (mL/kg/h); RER, respiratory exchange ratio (*V*CO_2_/*V*O_2_).

a Significant increase over vehicle levels (*P* < 0.05);

b Significant main effect of CR (*P* < 0.05).

## Discussion

Three weeks of food restriction induced marked adaptive thermogenesis in both resting and nonresting EE, where suppression of nonresting EE accounted for over one‐third of the total decrease in EE (Fig. 1). Food restriction suppressed physical activity levels (Table 1), and it also decreased the caloric demand of physical activity, even when activity levels were held constant and the change in body composition was accounted for (Fig. 1G). This was reflected in lower activity thermogenesis as assessed through muscle heat dissipation during low‐to‐moderate‐intensity activity. These data highlight the relevance of reduced physical activity EE – stemming from the dampening of both the amount and energetic cost of activity – to the adaptive thermogenesis seen during energy restriction; in turn, diminished muscle heat dissipation points to thermogenic mechanisms as potential mediators (Gamu et al. 2014). This is the first report of reduced muscle NETO, indicating lower SNS drive to skeletal muscle after 3 weeks of food restriction (Fig. 2), an effect not seen during short‐term energy restriction (Dulloo et al. 1988). Given the importance of skeletal muscle to both resting and activity EE, (Zurlo et al. 1990; Gallagher et al. 1998) this low SNS drive could contribute to both the resting and nonresting aspects of adaptive thermogenesis. Despite the suppression in EE, muscle SNS drive, and activity‐associated muscle thermogenesis (Fig. 1H), the ability of a central MC4R agonist to augment activity‐related EE remained intact during CR (Fig. 3), as did the ability of moderate‐intensity activity to increase muscle thermogenesis. Altogether, the data implicate diminished activity thermogenesis as one culprit in the adaptive thermogenic response that counteracts weight loss during food restriction, while the capacity of at least some stimuli to increase thermogenesis is maintained. This provides potential avenues to counter adaptive thermogenesis and promote continued weight loss and weight maintenance through targeting physical activity EE and skeletal muscle thermogenesis.

Our results demonstrating a reduction in daily EE due to weight loss (Fig. 1) are consistent with findings from laboratory animals (Ravussin et al. 2011; De Andrade et al. 2015) as well as clinical studies (Leibel et al. 1995; Goldsmith et al. 2010) conducted under different conditions. After a 19% reduction in rats' body weight, total EE was markedly suppressed (42%). Although little variance in adaptive thermogenic response was found in this outbred population (Fig. 1), response to food restriction could differ based on dietary composition or obesity propensity, as has been found in human populations (Reinhardt et al. 2015). In humans, when obese individuals maintain a reduced body weight, nonresting EE is suppressed proportionally more than resting EE as the magnitude of weight loss increases (Leibel et al. 1995; Rosenbaum and Leibel 2016). In rats, the CR‐induced adaptive thermogenesis in resting and activity‐associated EE was reflected in lower baseline and activity‐related temperature of the hind limb gastroc muscle (Fig. 1H); that muscle thermogenesis during moderate‐intensity activity was spared relative to resting activity thermogenesis may also suggest different mechanisms of induction (e.g., for skeletal muscle (De Andrade et al. 2015)). Elevated muscle thermogenesis is accompanied by lower locomotor efficiency in a rat model of leanness (Gavini et al. 2014) as well as after central activation of hypothalamic melanocortin receptors (Gavini et al. 2016). This implicates central modulation of skeletal muscle energetics through thermogenic mechanisms in the maintenance of leanness through enhanced activity EE. Indeed, muscle makes a substantial contribution to diet‐induced adaptive thermogenesis (van Baak 2008; den Berg et al. 2011; Gamu et al. 2014), so targeting muscle thermogenic mechanisms may prove to be an effective strategy to promote weight maintenance.

Positive and negative energy balance induce compensatory adjustments in autonomic nervous system function to oppose weight change (Aronne et al. 1995; Rosenbaum et al. 2000). Accordingly, decreased SNS outflow accompanies the suppressed activity EE seen after energy restriction‐induced weight loss in people (Rosenbaum et al. 2005). Though less attention has been focused on autonomic modulation of muscle compared to adipose tissue, some evidence suggests that SNS outflow to muscle participates in systemic metabolic effects (Gavini et al. 2014, 2016; Zhu et al. 2014). The hypothesis linking suppressed muscle work efficiency to changes in autonomic control would be strengthened by direct evidence of diminished SNS drive to muscle in the face of negative energy balance. Short‐term (2 days) fasting in rats decreases SNS drive to brown fat and heart, as measured using NETO, but had no detectable effect on skeletal muscle (Dulloo et al. 1988). Because the relative suppression of human nonresting EE increases to a greater extent with the severity of weight reduction compared to resting EE (Leibel et al. 1995), it stands to reason that exposure to a more extended or severe CR may also affect SNS drive to muscle. Indeed, here we demonstrate for the first time that rats subjected to 3 weeks of food restriction showed significantly lower NETO in skeletal muscle (Fig. 2). As expected, based on the importance of SNS‐stimulated lipolysis (Bartness and Song 2007) during CR to support the use of lipids as fuel (reflected in lowered RER; Table 1), white fat (EWAT) showed much higher NETO in the calorie‐restricted rats (Fig. 2D), consistent with other studies (Brito et al. 2008). The CR‐induced suppression of heart NETO is similar to what is seen after a short‐term fast in rats (Dulloo et al. 1988) and is consistent with altered SNS control of heart with weight reduction in humans (Aronne et al. 1995). This system‐specific impact of 50% CR on SNS outflow also implicates a role for neural catecholamines, rather than circulating hormones alone which would be expected to exert similar effects across organs. Altogether, these data point to system‐specific autonomic adaptations during energy restriction that differ according to the role of each system in energy conservation and metabolic fuel regulation, according to the demands placed on each system based on the severity of energy restriction. For muscle, the data support a hypothetical model in which, upon prolonged energy restriction, the brain reduces SNS outflow to skeletal muscle, inducing adaptations in muscle work efficiency, thereby suppressing muscle thermogenic processes. Evidence also supports adrenergic modulation of muscle thermogenic processes (Astrup et al. 1985; Schertzer et al. 2005; van Baak 2008; Bueno et al. 2010; den Berg et al. 2011; Pant et al. 2016). To some extent, this adaptive thermogenesis can be countered through activation of the brain melanocortin system, which increases not only activity EE and muscle thermogenesis but also SNS outflow to muscle (Gavini et al. 2016).

Because of the importance of brain melanocortin peptides and receptors (Song et al. 2005, 2008; Sohn et al. 2013) in autonomic control (Brito et al. 2007) and in the modulation of muscle energetics (Tanaka et al. 2007; Toda et al. 2009, 2013; Gavini et al. 2016), we hypothesized that the adaptations in activity EE could originate with altered responsiveness of this pathway. Our data did not support this hypothesis, however. As shown in Figure 3, activation of MC4R in the VMH increased EE during controlled treadmill walking, and activity EE decreased with CR, as predicted. The ability of central MC4R activation to augment activity EE above baseline values was preserved after CR, however (Fig. 3). This suggests that the suppressed activity EE, and likely the lower muscle thermogenesis and SNS drive, was not due to the inability of brain melanocortins to stimulate this brain‐SNS‐muscle pathway during CR. It is more likely that an upstream mediator is changed during CR, for example leptin, or the activation or suppression of this pathway by changes in endogenous release of melanocortin peptides or agouti‐related peptide (AgRP) (Li and Davidowa 2004; Cansell et al. 2012; Krashes et al. 2014), an MC4R inverse agonist. Though a direct comparison was not made here, activation of VMH MC4R receptors induced a different response than the mixed agonist melanotan II (MTII) in that MTII stimulated a higher magnitude change in EE and also lowered RER (Gavini et al. 2016). While energy restriction suppresses baseline levels, the ability to augment locomotor efficiency and muscle thermogenesis – through either activation of MC4R or moderate‐intensity activity – is preserved (Fig. 1H, Fig. 3), offering a potential target for interventions to combat the adaptive thermogenesis. Agents such as the MC4R agonist setmelanotide (Kuhnen et al. 2016) may impact muscle thermogenesis through central mechanisms. The intensity of physical activity also needs to be considered. Calorie restriction predominantly impacts efficiency and thermogenesis during low‐intensity activity in rats (Fig. 1H); similarly, in people subjected to weight reduction, muscle work efficiency is increased during cycle ergometry at 10W of power but not at 25W (Rosenbaum et al. 2003, 2005).

Three weeks of food restriction not only suppressed resting metabolism, but also decreased nonresting energy use by nearly half. The nature and timing of this suppression is comparable to what humans experience while maintaining a reduced weight (Rosenbaum et al. 2003, 2005; Camps et al. 2015; Rosenbaum and Leibel 2016). Suppression of nonresting EE, as well as resting EE, could potentially stem from lower SNS drive to skeletal muscle seen after 3 weeks of CR (Fig. 2) but not after a 2‐day fast (Dulloo et al. 1988). One outcome of the lower sympathetic drive to muscle during energy restriction could be the decreased muscle thermogenesis and EE seen during low‐intensity physical activity. The ability of central MC4R activation to enhance activity‐related EE is preserved during CR, however. Altogether, these and other studies suggest that reversal of adaptive thermogenesis in nonresting, activity‐related EE might be achieved through counteracting the changes in sympathetic drive to muscle and muscle thermogenic mechanisms, and highlight the potential of the MC4R‐driven brain‐muscle pathway in achieving this end.

## Conflict of Interest

CM Novak is funded by a Mid‐Career Research Grant funded by Novo Nordisk from The Obesity Society. The authors have no other conflicts of interest to disclose.
